# Long Non-Coding RNAs as Emerging Regulators of Pathogen Response in Plants

**DOI:** 10.3390/ncrna8010004

**Published:** 2022-01-11

**Authors:** Yashraaj Sharma, Alok Sharma, Madhu, Shumayla, Kashmir Singh, Santosh Kumar Upadhyay

**Affiliations:** 1 Department of Botany, Panjab University, Chandigarh 160014, India; yashraajsharma1@gmail.com (Y.S.); sharmaalok0001@gmail.com (A.S.); mds.3333stu@gmail.com (M.); shumaylasaifi5@gmail.com (S.); 2 Department of Biotechnology, Panjab University, Chandigarh 160014, India; kashmirbio@pu.ac.in

**Keywords:** abiotic stress, biotic stress, fungal, lncRNAs, plant development, viral

## Abstract

Long non-coding RNAs (lncRNAs) are transcripts without protein-coding potential that contain more than 200 nucleotides that play important roles in plant survival in response to different stresses. They interact with molecules such as DNA, RNA, and protein, and play roles in the regulation of chromatin remodeling, RNA metabolism, and protein modification activities. These lncRNAs regulate the expression of their downstream targets through epigenetic changes, at the level of transcription and post-transcription. Emerging information from computational biology and functional characterization of some of them has revealed their diverse mechanisms of action and possible roles in biological processes such as flowering time, reproductive organ development, as well as biotic and abiotic stress responses. In this review, we have mainly focused on the role of lncRNAs in biotic stress response due to the limited availability of knowledge in this domain. We have discussed the available molecular mechanisms of certain known lncRNAs against specific pathogens. Further, considering that fungal, viral, and bacterial diseases are major factors in the global food crisis, we have highlighted the importance of lncRNAs against pathogen responses and the progress in plant research to develop a better understanding of their functions and molecular mechanisms.

## 1. Introduction

Enhanced knowledge of the involvement of non-protein-coding regions of DNA in regulatory functions has provided remarkable progress in elucidating the important roles of non-coding molecules. The non-coding regions of the genome are not involved in any protein coding. The main focus on the protein-coding region of DNA delayed the discovery and the functional elucidation of the non-protein-coding regions of DNA and misidentified it as a “junk DNA” [1]. The non-coding regions of DNA are transcribed into a large number of transcriptional units known as non-coding RNAs (ncRNAs). These ncRNAs were initially considered to be trash DNA, but recently, they have become a key component of various regulatory processes [2]. High-throughput sequencing and expression profiling have given the limelight to these transcriptional units after decades of existential crisis [3].

Non-coding RNAs can be classified into two broad categories: housekeeping and regulatory ncRNAs. Structural ncRNAs such as tRNA, rRNA, snRNA, and snoRNA are grouped into housekeeping ncRNAs and are constitutively expressing in the cell [4]. Based on their length, small RNAs (sRNAs such as miRNA, siRNA, and piRNA) and lncRNAs are various types of regulatory ncRNAs. Earlier, researchers had emphasized the key role of sRNA in the regulation of gene expression at the transcriptional and post-transcriptional level and considered lncRNAs to be “transcriptional noise”. However, recent studies on model plants have revealed several important roles of lncRNA in various plant development processes, for instance, regulation of photomorphogenesis by red light, flowering, and biotic and abiotic stress responses [5,6,7,8,9]. The first eukaryotic lncRNA was H19, identified in the mouse [10]. *GmENOD90* from *Glycine max* was the first identified plant lncRNA using RNA sequencing and in vitro based analysis [11]. Due to high-resolution transcriptomic analysis, the identification of lncRNAs in plants has blossomed and caught up with the mammalian research field in a few years [11]. Long non-coding RNAs are transcribed by RNA polymerase II, similarly to mRNAs, and processed through 5′-capping and 3′ poly (A) addition. In addition to RNA Pol II, they are also transcribed by polymerase IV/V in plants [12,13] and processed through splicing or non-splicing and polyadenylation or non-polyadenylation. Long non-coding RNAs can be broadly classified on the basis of their genomic location as: (i) long intergenic ncRNAs (lincRNAs), (ii) long intronic ncRNAs (incRNAs), and (iii) natural antisense transcripts (NATs) [14]. Their subcellular localization can be nuclear or cytoplasmic. The lincRNAs and incRNAs are transcribed from intergenic and intronic regions of DNA, respectively, while NATs originate from associated genes of complementary DNA strands [15]. Apart from the above groups, there is one more group of lncRNA, which are less in abundance and are transcribed from back-spliced exons, known as circular non-coding RNAs (circRNAs). Some lncRNAs are transcribed by RNA polymerase II from regions of essential DNA elements such as promoter and enhancer, for instance, PROMPT and eRNAs. These RNAs are short lived with rapid turnover rates and targeted by the RNA exosome in the nucleus, indicating their significance in gene regulation [16]. The ideally known function of lncRNAs might be in the regulation of transcription as “riboregulators.” Moreover, the molecular mechanism of lncRNAs can be depicted in several ways; they can act as signals and decoys of miRNAs or a competitor of pre-mRNAs in alternate splicing, as a guide in directing RNP (ribonucleoprotein) complex to specific targets, and as scaffolds in the recruitment of complex protein molecules [17].

## 2. Origin and Database Development of Plant lncRNAs

The mechanisms of plant adaptation to different stresses that we see today are the result of a joint venture among plants, microorganisms, and different types of environmental conditions. Plants have encountered various biotic and abiotic stresses during the long course of evolution and these interactions have been recorded in the form of intricate molecular mechanisms. These mechanisms developed over time and help in many developmental processes by surviving and sustaining harsh conditions such as mechanical and biological stresses. A genome’s transcriptional inventory consists of coding as well as non-coding RNAs, and the latter is said to be equally contributing to the plant’s complex adaptations processes. Non-coding RNAs (ncRNAs) have emerged as crucial bioactive molecules that contribute to genome and phenotypic diversity. Only around 2% out of 90% of the transcribed RNAs from the eukaryotic genome result in the production of proteins [18,19]. Next-generation sequencing (NGS) technology advancements have been critical to finding ncRNAs in plants, in combination with homology-based and/or experimental techniques [20,21]. High-throughput sequencing has made major advances in understanding the biology of lncRNAs. Plant lncRNAs remain a mystery, despite new findings that provide insight on their functions and methods of action. Moreover, the origins and roles of these ncRNAs have been reported to be diverse [22]. Existing transposable elements (TEs), random hairpin configurations, pseudogenization of protein-coding sequence, and DNA repeat are some prominent explanations for the origins of various ncRNAs such as miRNA, siRNA, piRNA, and lncRNA [23,24,25,26].

Despite being poorly conserved, sequence conservation among subsets of lncRNAs across the species can be found [27]. On exploring biological significance and evolution across 23 plant species, low sequence conservation at the transcript level among the majority of lncRNAs has been observed [28]. Conservation studies of lncRNAs to predict their possible functions are difficult because of less availability of tools and datasets. However, some databases have been developed recently and are made available to researchers to decipher their phylogenetic relationship, expression pattern, and molecular interactions. Plant specific databases such as PlncRNAdb, PLncDB 2.0, CANTATAdb 2.0, GreeNC, TAIR10, PNRD, and PlantNATsDB, along with databases containing information from the plant as well as other organisms (e.g., RNA central, EVLncRNAs, lncRNAdbv2.0, and NONCODE v4) are available to make more in-depth inferences on the lncRNAs [29]. Some of the databases with their features for the identification of plant lncRNAs have been listed in the table below (Table 1).

## 3. Involvement of lncRNAs in Various Biological Processes

**Figure 1:**
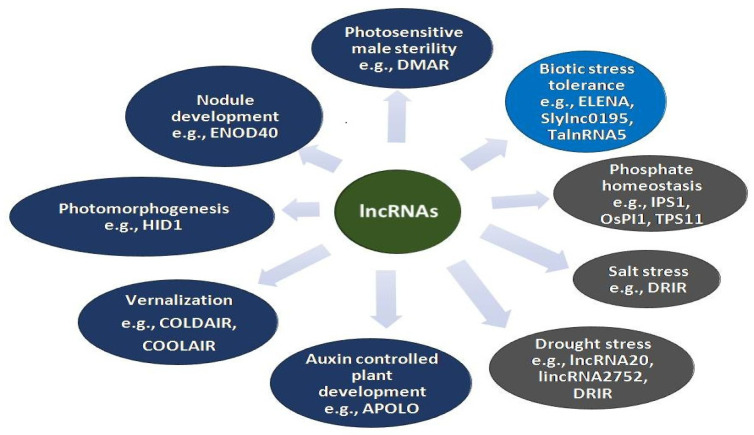
Roles of some of the functionally characterized lncRNAs in different biological processes.

Long non-coding RNAs control the gene regulation processes mostly at the level of mRNA processing, editing, and turnover [36]. However, some lncRNAs also display gene regulatory functions by post-transcriptional modifications. They mediate regulatory functions by binding to DNA/RNA either through *cis*-acting or *trans*-acting sequences. Various lncRNAs have been identified in plants coupled with the defense responses related to plant immunity and adaptation to environmental conditions. Many lncRNAs have been documented using *in silico* analysis, whole-genome and RNA sequencing in different plants such as *Arabidopsis thaliana* [37], *Triticum aestivum* [38], *Oryza sativa* [39], *Zea mays* [40], *Medicago truncatula* [41], *Vitis vinfera* [42], etc. Ample reports have been published on the possible functions and molecular mechanism of lncRNAs despite being less explored and functionally characterized [43]. Their mode of action and targeting mechanism differ in different biological processes. Most of the reported lncRNAs are stress responsive [44]. However, their roles have also been reported in other plant development processes (Figure 1). The lncRNAs involved in the regulation of flowering time are the most diverse studied group of lncRNAs in model plants. FLOWERING LOCUS C (FLC), a key regulator of flowering time in *Arabidopsis* is epigenetically regulated by lncRNAs *COLD INDUCED LONG ANTISENSE INTRAGENIC RNAs (COOLAIR)* and *COLD-ASSISTED INTRONIC NON-CODING RNA (COLDAIR)* [20,45,46]. *COOLAIR* and *COLDAIR* help in recruiting PHD-PRC2 complex enabling histone modification of FLC. Some lncRNAs such as *EARLY NODULIN 40* (*ENOD40*) and *AUXIN REGULATED PROMOTER LOOP* (*APOLO*) are involved in nodule development and polar auxin transport, respectively. *ENOD40* participates in the nodule development in the leguminous plants by relocalization of RNA binding protein 1 (RNP1) from nuclear speckles to cytoplasmic granules [47]. *APOLO,* an intergenic lncRNA, is transcribed by RNA polymerase II and V, this dual *APOLO* transcription directs the chromatin loop dynamics modulating the expression of neighbor *PID* gene that plays an important role in the regulation of polar auxin transport [48,49]. Several lncRNAs have been reported to be involved in regulating various kinds of challenges related to abiotic stresses. For instance, under phosphate starvation conditions, a lncRNA *Induced by phosphate starvation* 1 (*IPS1*) is expressed in *Arabidopsis* which promotes phosphate uptake and accumulation. It acts as an endogenous target mimic (eTM) for miR399, which is a repressor of *PHOSPHATE2* (*PHOS2*). PHOS2 is responsible for encoding ubiquitin-conjugating (E2) enzymes, and its repression enhances phosphate uptake and accumulation [50]. Some lncRNAs are responsive to light such as *HIDDEN TREASURE 1* (*HID1*). It negatively regulates the expression of the *PHYTOCHROME-INTERACTING FACTOR* (*PIF3*), a transcription factor that is the main repressor in photomorphogenesis resulting in the hypo-photomorphogenic response under red light conditions [5,51]. Some lncRNAs regulate photoperiod-sensitive genetic male sterility modulating reproductive organ development. The reduced transcript level of long day-specific male fertility-associated RNA (*LDMAR*) under long-day conditions causes programmed cell death at the time of anther development in rice leading to male sterility [52,53].

## 4. Roles of lncRNAs in Various Biotic Stress Responses

**Figure 2:**
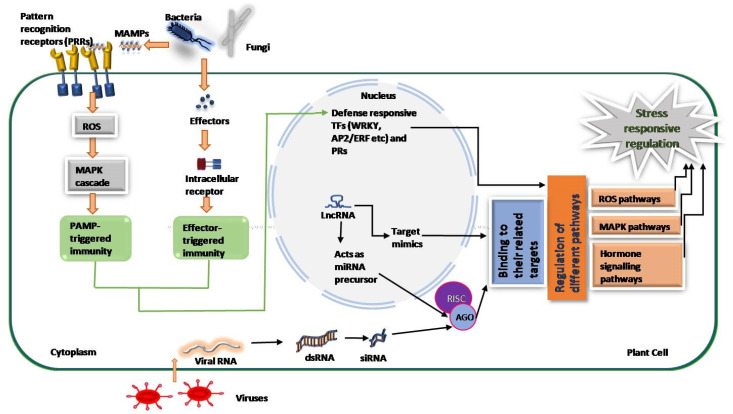
A general model of stress-responsive regulation by regulatory lncRNAs. After stress signal perception, PAMP-triggered immunity (PTI) is activated through the production of signal transducers such as reactive oxygen species (ROS). Pathogen-specific effector-triggered immunity (ETI) is activated by NB-LRR resistance (R) genes after effectors such as the virulence factor of pathogens, enter into the plant cells. PTI and ETI both lead to activation of defense-related pathways. Long non-coding RNAs play important regulatory functions in various plant defense mechanisms either by acting as a precursor of miRNAs and siRNAs or as a miRNA target mimic. (NB-LRR, nucleotide-binding leucine-rich repeats; MAPK, mitogen-activated protein kinase; TFs, transcription factor; RISC, RNA-induced silencing complex; AGO, argonaute proteins).

Plants have evolved through sets of defense mechanisms to mitigate different diseases effectively. Plant cells respond to pathogen attacks after pathogen recognition, triggering downstream signaling networks at the molecular level to arrange transcriptional machinery [54]. A minimal effort has been made in identifying and annotating the lncRNAs related to biotic stresses. Therefore, the molecular mechanisms of some lncRNAs in three major biotic stresses have been highlighted below (Table 2 and Figure 2).

### 4.1. Long Non-Coding RNAs against Fungal Infection

Commercially valuable crops such as wheat, rice, tomato, and cotton can be severely damaged by fungal diseases. Powdery mildew (PM) and stripe rust in wheat caused by *Blumeria graminis f.* sp. *tritici* (Bgt) and *Puccinia striiformis f.* sp. *tritici* (Pst), respectively, are such examples. Rice blast is another example of such a destructive disease caused by *Magnaporthe oryzae* and resulting in remarkable yield loss. The molecular studies of these diseases can provide better insights for developing pathogen resistance strategies.

A comparative expression profile analysis of two cultivars of wheat (PM-susceptible JD8 and PM-resistance JD8-pm30) in response to powdery mildew (PM) infection has revealed expression patterns of 71 lncRNAs in a tissue-specific manner [58]. It was found that some (TapmlnRNA5, TapmlnRNA8, and TapmlnRNA19) differentially expressing lncRNAs in different tissues were precursors of miRNA having stable hairpin structures. The tissue dependent expression of these lncRNAs in response to Bgt infection indicates their role in the development and regulation of biotic stress [58].

By investigating changes in transcriptionally active regions (TARs) (both TARs antisense to overlapping or adjacent genes and intergenic TARs) using a strand-specific RNA-seq approach, 15 lncNATs and 20 lincRNAs were identified in *Arabidopsis thaliana* against *Fusarium oxysporum* infection [60]. Functional characterization of some lncRNAs using knockout or knockdown in Arabidopsis plants have revealed evidence of lncRNAs role against disease development. For instance, novel intergenic TAR-191 and TAR-197 induced upon *F. oxysporum* infection were attenuated in the RNA interference (RNAi) line and T-DNA insertion knockout lines, respectively, and these lines were showing significant disease development [60].

Intriguingly, there was a negative correlation between co-induction of transcripts from the *At2g30770* gene and its lncNATs after *F. oxysporum* infection. The *At2g30770* gene encodes CYP71A13, an essential P450 enzyme involved in the biosynthesis of an essential phytoalexin called camalexin which plays important role in disease resistance. In the core promoter of this gene, TCA-element and TC-rich repeat are found which are responsive to salicylic acid and stresses, respectively, suggesting the presence of shared TFs binding sites and pathogen responsive elements in the promoter regions [60].

*Phytophthora infestans* which is the causal agent of late blight (LB) in tomatoes causes serious economic loss worldwide in field-grown tomatoes and is a major threat to its production. A comparative transcriptomic analysis of resistant (*Sp*) and susceptible tomatoes lines (*Slz*) against *P. infestans* infection identified a total of 1037 differentially expressed genes (DEGs) and 688 DElncRNAs (DELs) [64]. After analyzing co-localization and expression between DEGs and DELs, *lncRNA16397,* a lncNAT of the glutaredoxin gene *SlGRX22,* was identified, which regulates expression of *SlGRX22*. GRXs are glutathione-dependent disulfide oxidoreductases involved in oxidative stress response in plants [65]. The *lncRNA16397* and *SpGRX* overexpressing plants showed fewere LB symptoms, depicting their roles in disease resistance. *SpGRX* functions by reducing ROS accumulation and alleviating injury in cell membrane eventually enhancing resistance in tomato against *P. infestans*.

A genome-wide investigation carried out in susceptible *Vitis vinifera* in response to obligate biotrophic phytopathogens *Erysiphe necator* and *Plasmopara viticola,* causing agents of PM and DM (Downey mildew), respectively, identified 71 PM and 83 DM-responsive lncRNAs [66]. A co-expression analysis revealed that 52 PM responsive lncRNAs were co-regulating with 33 protein-coding sequences (CDS) and 22 DM-responsive lncRNAs were found to be co-expressed with 127 CDS. Further, a Gene Ontology (GO) enrichment analysis revealed the functional annotation of these CDS sequences, and highlighted the putative role of the co-expressed lncRNAs in plant defense response against the PM and DM infection [66]. Similar results were also previously found in *V. vinifera,* in response to *Botrytis cinerea* (grey mold (GM)). A co-expression analysis of 47 GM-responsive lncRNAs and 179 CDS revealed potential interaction between them and the role of lncRNAs in defense response against GM infection [67].

Plants have evolved the innate immune system to counter various harmful pathogens such as fungi. They can respond against a range of pathogens carrying pathogen-associated molecular patterns (PAMPs) and effector molecules [68]. PAMP-triggered immunity (PTI) acts as the first line of plant innate immunity against a pathogen attack. PAMPs are recognized by trans-membranous pattern recognition receptors (PRRs), which trigger a weak immune response called PTI [69,70]. The second line of plant innate immunity is effector-triggered immunity (ETI), which is activated upon the recognition of pathogen molecules called virulence (Avr) effectors [71]. These highly variable effector molecules trigger robust hypersensitive reactions mediated by highly polymorphic plant resistance (R) proteins. The innate immune system of plants, via these two layers, counters a pathogen attack by activating many defense-related genes by various signaling pathways [72,73,74,75,76]. The lncRNAs may alter the expression of those defense-related genes at the transcriptional and post-transcriptional levels to mitigate the pathogens attack. However, their role in adaptation and specificity against pathogens and their interaction with lncRNAs is yet to be deciphered [54]. Transcription factors (TFs) such as NAC, AP2/ERF, WRKY, and C2H2 have been reported to be involved in the regulation of plant response against pathogens. A co-expression analysis of some lncRNAs with these neighboring TFs has suggested a co-regulation relationship with adjacent protein-coding genes, predicting regulatory roles of lncRNAs in both positive and negative ways. Many fungi-responsive lncRNAs have been identified and their functions have been predicted with the help of technologies such as genome-wide microarray analysis and SBS sequencing, supporting the involvement of lncRNAs in the basal defense mechanism of plants. Most of the functional cues of lncRNAs based on bioinformatical analysis have suggested that lncRNAs can potentially interact with other classes of ncRNAs including small non-coding RNAs such as miRNAs [66,67]. In addition, techniques such as RNAi and T-DNA insertion have suggested their role by acting as either a precursor of miRNA or miRNA mimics, yet their functional network is to be deciphered.

### 4.2. Long Non-Coding RNAs against Viral Infection

Plant viruses cause significant economic losses in a wide range of crops. Virus infections cause symptoms such as necrosis, yellowing, leaf spot, mosaic color, and abnormal growth in plants. These viruses could possess either DNA or RNA as their genetic material. However, DNA viruses are the less common agent of plant diseases and mostly the single or double-stranded RNA viruses infect plants by utilizing RNA-dependent RNA polymerase (RDRP) activity. An RNA virus usually replicates in the cytoplasm with few exceptions such as retroviruses and negative single-stranded RNA (ssRNA) viruses.

**Figure 3:**
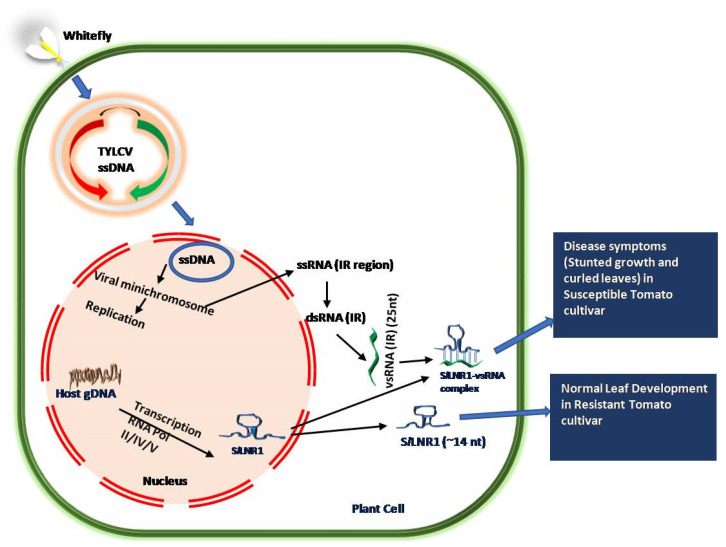
Mechanism of the pathogenesis of the TYLCV virus in susceptible and resistant tomato cultivar. Binding of vsRNA in susceptible cultivar leads to disease symptoms, while deletion in S*l*LNR1 sequence in resistant tomato cultivar shows no disease symptoms.

Generally, pathogen recognition activates a cascade of signaling pathways governing defense response. However, recently, the roles of lncRNAs involved in defense responses have been emerging. An expression analysis of lncRNAs indicated that several lncRNAs such as lincRNA and lncNATs were differentially regulated in response to the tomato yellow leaf curl virus (TYLCV) infection in TYLCV-resistant cultivar CLN2777 [62]. TYLCV belongs to the DNA Geminivirus containing a single-stranded circular DNA molecule in its genome. The lincRNAs such as *slylnc0048*, *slylnc0049*, *slylnc0483*, *slylnc0531*, and *slyinc0934* were found to be upregulated and *slylnc0475*, *slylnc0476*, *slylnc0673*, and *slylnc1052* were downregulated in an expression analysis, suggesting their role in defense response against viral infection. Later, the lncRNA S*l*LNR1 from tomato (*Solanum lycopersicum* L.) was reported to be involved in plant growth and leaf development. A viral small interfering RNA (vsRNA) derived from the intergenic region (IR) of TYLCV is almost perfectly complementary with long non-coding RNA, S*l*LNR1. The non-coding IR sequence of intergenic regions consists of 25 nucleotides, which are required for replication and transcription within the host cells. The vsRNA induces silencing of S*l*LNR1 in TYLCV-susceptible tomato cultivar by RNAi and its downregulation causes stunted plant growth and curled leaf phenotypes [77]. A deletion in the 25 nt IR sequences has provided resistance to vsRNA-directed repression, as in the case of TYLCV-resistant cultivar CLN2777 (Figure 3). This is a good example of post-transcriptional gene silencing in which S*l*LNR1 is targeted by IR derived siRNA.

Studies have predicted the possibility of a complex gene-regulating relationship between lncRNA and mRNA. Rice black-streaked dwarf virus (RBSDV) is a non-enveloped RNA virus transmitted through small brown planthopper. The transcriptomic profile of RBSDV-infected rice plants has revealed a total of 1342 differentially expressed (DE) mRNAs and 22 differentially expressed lncRNAs [78]. A co-expression network analysis of these DEmRNAs and DElncRNAs has shown a possible sign of the presence of a gene regulatory network in the plant–pathogen interaction pathways. An RBSDV infection results in the expression of many lncRNAs regulating the expression of several genes beneficial in plant defense mechanisms, as well as in viral pathogenesis. Genes beneficial to host plants in defense response include genes encoding for LRR domain-containing proteins, ubiquitin-mediated proteasomal degradation proteins, and calmodulin-like proteins, as well as genes related to flavonoid biosynthesis. The genes beneficial in viral pathogenesis involve genes related to hormone signaling and biosynthesis. The molecular mechanisms of a very few lncRNAs have been understood in viral response until now. Therefore, in addition to the PTGS through siRNAs, some lncRNAs also act as endogenous target mimics (eTM) for miRNAs in plants. For instance, silencing of slylnc0195 by viral-induced gene silencing (VIGS) has resulted in the accumulation of virus in slylnc0195-VIGS *Nicotiana benthamiana* plants showing miR166 as its putative targets and its functioning via miRNAs target mimics [62] and lncRNALNC_1497 which acts as miRNA mimics of MIR4995-p5_Iss19GC and regulates NAC gene (Cla010201) in cucumber green mottle mosaic virus (CGMMV) infection [63].

### 4.3. Long Non-Coding RNAs against Bacterial Infection

In addition to fungi and viruses, bacteria are another major threat to plants, which causes yield loss by means of various diseases. The role of lncRNAs in bacterial disease resistance is still less explored as compared with other known pathogens. However, a few studies have demonstrated the involvement of lncRNAs in bacterial disease resistance [55,56,57]. Bacterial canker disease of kiwi fruit is caused by the *Pseudomonas syringae* pv. *actinidiae* (*Psa*) and shows variable symptoms such as dark brown spots on leaves and cankers on the stem. The kiwi fruit responds through a variety of immune processes against the *Psa* pathogen. The upregulation of lncRNAs and their interaction with various signaling and defense-related genes has been reported in Psa-infected kiwi fruit. The lncRNAs were predicted to provide immunity to plants by playing their roles in systematic acquired resistance (SAR), salicylic acid-mediated signaling pathway, and chitin catabolic processes [54]. Plants activate the diverse defense responses in turn of microbe signal detection through PRRs. These responses include the production of assorted antimicrobial compounds and PR proteins [79,80]. The PR group of proteins are important due to their antimicrobial activities [56]. Recently, Seo et al. identified a lncRNA named *ELF18-INDUCED LONG-NONCODING RNA1* (*ELENA1*) in the *Arabidopsis thaliana*. Overexpression of *ELENA1* increased the degree of resistance against *Pseudomonas syringae* pv. tomato DC3000 by upregulating the PR1 proteins [55].

*Xanthomonas oryzae* pv. *oryzae* (Xoo) causes bacterial leaf blight disease of rice plants. It is considered to be the most harmful disease of rice, which is responsible for rice destruction throughout the world. Yu et al. (2020) carried out the strand-specific RNA sequencing of *Xanthomonas oryzae* pv. *Oryzae* (Xoo) infected leaves of rice at 2, 6, 12, and 24 h of post-inoculation. In their work, they identified a total of 567 differential expressed lncRNAs. Further, these lncRNAs showed their interaction with a variety of stress-related mRNAs, which provided insight into their roles in plant defense pathways. The enhancer trap system approach was used to develop the mutant rice plants having overexpressed *ALEX1* (a leaf expressed and Xoo-induced lncRNA 1). Jasmonic acid (JA) is an important phytohormone known to play a vital role in rice–Xoo interactions. The JA and salicylic acids are reported to enhance the various defense pathways by regulating the different PR proteins. ALEX1 was found to be responsible for the activation of the JA pathway. The participation of ALEX1 in JA signaling activation has been confirmed by various findings such as upregulation of JA interacting genes, root and shoot growth inhibition, and increased level of jasmonates in mutant rice plants [57]. Collectively, these findings show the connection of lncRNAs in bacterial disease resistance by mediating the various signaling pathways; however, the exact mechanism of action is still ambiguous and needs to be explored in future studies.

## 5. Conclusions

Rapid identification of novel lncRNAs and their functional characterization has become possible due to recent advances in high-throughput sequencing and computational biology. It has revealed the multifaceted regulatory function of lncRNAs in composite regulatory pathways, playing a vital role not just in plant immune response but also in several plant development processes, hormone signaling, and pathogenesis. Despite their known roles as miRNA and siRNA precursors, and miRNAs mimic, the specific role of lncRNAs in defense-related signaling pathways is yet to be deciphered. However, the specific role of some of the lncRNAs in different stresses has been identified in model plants such as *Arabidopsis* and tomato, using techniques such as RNAi and virus-induced gene silencing. The synteny relationship among crop plants and model plants can give a major boost to the functional characterization of novel lncRNAs of crop plants. In this review, we have summarized the mode of action and functions of lncRNAs related to some major biotic stresses. It is important to emphasize research about stress regulatory lncRNAs due to their significance in crop improvement programs. The molecular mechanisms regulating stress responses such as functional crosstalk among miRNA and lncRNAs are still unexplored and have significant importance in enhancing our understanding of stress tolerance. The ability of lncRNAs to mitigate regulatory and functional proteins at the transcriptional and post-transcriptional levels has flagged them as key players not only in the cellular and developmental processes but also in stress response. Therefore, we present lncRNAs as worthy candidates requiring attention in future research that will further strengthen our knowledge to overcome the global food crisis caused by pathogens.

## Figures and Tables

**Table 1:** Some databases available for the identification of plant lncRNAs.

Database	Features	Reference
Plant long non-coding RNA database	This database consists of >13,000 lincRNAs and associated epigenetic markers	[18]
Plant ncRNA database	It consists of 11 different types of ncRNAs of 150 plant species	[30]
Green non-coding database	It consists of data of 37 plant species and algae with more than 120,000 lncRNAs	[31]
The Arabidopsis information resource	It also consists of data of various noncoding RNAs	[32]
Araportll	It consists of annotated lincRNA, NATs, and various other ncRNAs	[33]
Plant natural antisense transcripts database	It consists of NATs annotated data along with expression of small RNA of 70 plant species	[34]
CANTATAdb	It consists of data of 45,000 lncRNAs of 10 model plant species	[35]

**Table 2:** Long non-coding RNAs related to the biotic stress response.

Pathogen	Associated Stress	lncRNA	Mechanism	Plant	Reference
Bacteria	Bacterial speck disease (*Pseudomonas syringae* pv. tomato DC3000)	Up- ELENA1	Directly interact with MED19a	*Arabidopsis thaliana*	[55]
	Bacterial canker (*Pseudomonas syringae* pv. *actinidiae*)	Up- TCONS_00202033, TCONS_0019494 & TCONS_00076221	Unknown	* Actinidi adeliciosa *	[56]
	Bacterial leaf blight (*Xanthomonas oryzae* pv. *oryzae*)	Up- ALEX1	Interacts with JA related genes	* Oryza sativa *	[57]
Fungal	Powdery mildew (*Blumeria graminis* f. sp. *tritici*)	Up- TalnRNA5, TapmlnRNA19	Precursor of miR2004	*Triticum aestivum*	[58]
	Powdery mildew (*Blumeria graminis* f. sp. *tritici*)	Up- TalnRNA9	Signal recognition particle 7S RNA variant 1	*Triticum aestivum*	[58]
	Powdery mildew (*Blumeria graminis* f. sp. *tritici*)	Up- TapmlnRNA2, TapmlnRNA7	Precursor of siRNA	*Triticum aestivum*	[58]
	White mold (*Sclerotinia sclerotiorum*)	Up- TCONS_00012499, TCONS_00004577, TCONS_00004034, TCONS_00009614, TCONS_00015411	Precursor of mi156	*Brassica napus*	[59]
	White mold (*Sclerotinia sclerotiorum*)	Up- TCONS_00006568, TCONS_00018692, TCONS_000017152, TCONS_00008591, TCONS_00001092	Precursor of mi169	*Brassica napus*	[59]
	Wilt disease (*Fusarium oxysporum*)	Up- TAR-66 (lincRNA)	Co-induction with neighboring defense-related gene	*Arabidopsis thaliana*	[60]
	Wilt disease (*Fusarium oxysporum*)	Up- TAR- 67,-191,- 197,-224	Unknown	*Arabidopsis thaliana*	[60]
	Stripe rust (*Puccinia striiformis* f. sp. *tritici*)	Up- TalncRNA18, 106	Unknown	*Triticum aestivum*	[61]
	Stripe rust (*Puccinia striiformis* f. sp. *tritici*)	Up & Dp at different dpi- TalncRNA73, 108	Unknown	*Triticum aestivum*	[61]
Viral	TYLCV Infection	Up- Slylnc0195	Target mimicry of miR166	*Solanum lycopersicum*	[62]
	TYLCVInfection	Dn- Slylnc1077	Target mimicry of miR399	*Solanum lycopersicum*	[62]
	CGMMV infection	Up- lncRNALNC_1497	Target mimicry of MIR4995-p5_Iss19GC	*Citrullus lanatus*	[63]

Up, upregulation; Dn, Downregulation; dpi, days post-inoculation; TAR, transcriptionally active region; TYLCV, tomato yellow leaf curl virus; CGMMV, cucumber green mottle mosaic virus.

## Data Availability

Not applicable.
